# Epidemiological, Clinical and Entomological Characteristics of Yellow Fever Outbreak in Darfur 2012

**DOI:** 10.3934/publichealth.2015.1.132

**Published:** 2015-03-25

**Authors:** Hamdi Abdulwahab Alhakimi, Omima Gadalla Mohamed, Hayat Salah Eldin Khogaly, Khalid Ahmad Omar Arafa, Waled Amen Ahmed

**Affiliations:** 1 Federal Ministry of Health, Public Health Institute & Epidemiology Department, Sudan; 2 Federal Ministry of Health, Epidemiology Department, Sudan; 3 Albaha University, Faculty of Applied Medical Sciences, Saudi Arabia

**Keywords:** Yellow Fever, outbreak, epidemiology, entomology

## Abstract

The study aims at analyzing the epidemiological, clinical and entomological characteristics of Darfur yellow fever epidemic. It is a descriptive, cross-sectional study. According to operational case definition, suspected yellow fever cases are included in case spread sheet with variables like age, sex, locality, occupation, status of vaccination, onset of symptoms, presenting symptoms, date of blood sampling and confirmation of diagnosis either by laboratory results or epidemiological link. Data about important entomological indices were collected by surveys conducted in 17 localities of 3 Darfur states (Central, West and south Darfur). All Darfur states (especially Central Darfur) have been affected by Yellow Fever outbreak. There is a need to review the non-specific case definition of Yellow Fever which seems to overwhelm the system during outbreaks with cases of other endemic diseases. The significant risk factors of this outbreak included male sex, adult age, outdoor occupation and traditional mining. The fatality rate was significantly associated with vaccination status. The highest fatality rate was recorded by children less than 2 years old (42.9%). Generally, increase in certain entomological indices was followed by increase in number of reported cases 7 days later. Central Darfur state was significantly higher in most studied entomological indices.

## 1. Introduction

World Health Organization (WHO) reported that; Yellow Fever endemic region in Africa is located approximately between 15° north to 15° south of the equator [1]. The number of YF cases increases since 1980s, which can be partially attributed to improved surveillance systems and better access to laboratory services [2].The high transmission of YF virus among human in Africa is associated with the presence of high density of vector in areas where a large numbers of unvaccinated populations found [3]. In Sudan, several outbreaks of yellow fever were reported from different parts of the country. The first reported outbreak was in 1940 in the Nuba Mountains and South Kordofan and the second outbreak was reported from the same area in 2005.

The yellow fever outbreak in this study occurred in Darfur states during the period October 2012–January 2013 [4].The unfavorable conditions in Darfur region, resulting from political instability and displacement of people, facilitated the occurrence of yellow fever outbreak. Regarding reported cases, this outbreak was huge with no similar yellow fever outbreak reported in Africa in the last 20 years [5]

Darfur is a region located in western Sudan. In January 2012, it was divided into five states namely: North Darfur, South Darfur, West Darfur, East Darfur and Central Darfur. It covers an area of 493,180 square kilometres and the population was estimated to be about 8.3 million in 2012 [6]. The international borders of Darfur region include: Central African Republic (CAR), Chad, Libya and South Sudan (since separation in July 2011). Internally, it has borders with North and South Kordofan states to the southeast; and with Northern State to the northeast.

The present study analyzed the epidemiological, clinical and entomological characteristics of Darfur YF outbreak and their association with fatality rate among suspected cases.

## 2. Materials and Method

Epidemiological and entomological surveys were conducted among suspected YF cases in 17 localities of 3 Darfur states (Central, West and south Darfur). The households were visited and the water storage containers were inspected for *AedesAegypti* (Larva and Pupa).

Data were extracted from YF line list developed by epidemiology department at Sudan Federal Ministry of Health. The data were anonymized and only ID numbers for suspected cases were available. The ethical clearance was obtained from Sudan Medical Specialization Board. The line list contained information about suspected cases included age, sex, locality, occupation, status of vaccination, date of onset of symptoms, presenting symptoms, date of taking the blood sample and the laboratory results either by ELISA (IgM) or PCR. Also, it was identified whether the diagnosis of YF was confirmed by laboratory or by epidemiological link. Attack rate (AR) and fatality rate (FR) were used throughout analysis as dependent variables

During the epidemic period, blood samples were taken from the patient suspected to have Yellow fever. Serum was separated and sent to National Public Health Laboratory (NPHL) for investigation. The result forms were filled and sent to the epidemiology department at federal level either as a hard copy or through an e-mail. All the laboratory results were reviewed and the data were exported into the YF line list.

The scarcity of laboratory reagents in NPHL resulted in delay of laboratory confirmation was officially notified by the National Public Health Laboratory on 26 October 2012. For the same reason, only 16% of blood samples were tested for YF because when one or two cases confirmed to be positive from a locality, then this locality was considered to be affected and no further blood samples were tested. The laboratory evidence of YF infection is through presence of YF specific IgM or detection of the YF antigen by ELISA or fragments of RNA genome by PCR.

It was claimed that the collected data depend on the cases satisfied the operational YF case definition modified from WHO standard case definition [7]. It had to include the following categories; suspected case which was any case with high grade fever and vomiting with negative blood smear for malaria; a probable case which was suspected case with jaundice, bleeding or week pulse rate and in severe cases oliguria; and confirmed case which was a suspected case that is laboratory-confirmed or epidemiologically linked cases (suspected cases had symptoms suggestive of YF and lived in same locality with laboratory confirmed case or cases. The laboratory evidence of YF infection is through the detection of YF specific IgM or by ELISA or detection of RNA genome by PCR

Entomological surveys were conducted in the affected localities in Central, South and West Darfur. Methods used during these surveys include knocking down, using trap for adult mosquitoes and searching for immature stages (larva and pupa) of Aedes species. The entomological indices (for larva and pupa) were calculated from the available daily reports. Entomological indices for each newly affected area at least10 households were inspected. The entomological indices were calculated for each locality as follows:

- The household index = number of positive houses for Aedes Aegypti/total number of houses inspected in the cluster;
- Container Index: = number of positive containers for Aedes Aegypti/total number of containers inspected in the cluster
- Breteau Index (BI) = number of positive containers for Aedes Aegypti/100 houses inspected in the cluster
- Pupal demographic index (PDI) = Number of pupa/Number of residences in the houses inspected

Area with a container index of ≤10% and household index <5% were considered safe for yellow fever transmission. While Breteau index of <5 indicate absence of yellow fever transmission [8].

The researcher used different types of statistical techniques so as to analyze the data. Statistical Package of social Sciences (version 20) used to analyze the collected data of all suspected cases of yellow fever which met the operational YF case definition that modified from the standard WHO case definition. Chi-square test used to associate between nominal variables. Logistic regression also used to model the relationship between certain clinical features and risk of death (fatality rate) among suspected cases. Correlations were assessed between incidence of YF cases and entomological data.

## 3. Results

**Figure 1.**
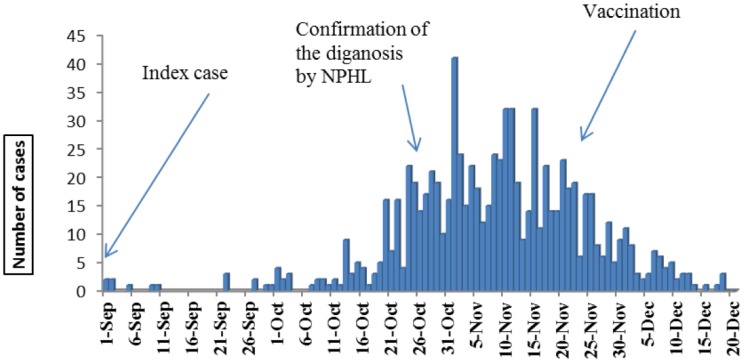
Distribution of YF reported cases by their date of onset from September to December 2012–2013 (*n* = 844).

A total of 849 YF cases were reported to Sudan Federal Ministry of Health as well as reported by World Health Organization WHO (see epidemic curve **(****Figure 1**) [9]). When reviewing the line list later on, it was found that 5 cases were duplicated. Despite of those 844 cases have been called suspected cases according to operational case definition modified by Federal Ministry of Health, only 58.6% of cases during statistical analysis have found to correspond for operational case definition as suspected cases. Therefore the analysis in this study was conducted on 844 reported cases (rather than suspected cases) which were hospitalized in treatment centres distributed in different Darfur states during the outbreak.

The socio-demographic characteristics of the YF reported cases are showed in the following table (**Table 1**).

**Table 1.** Socio-demographic characteristics of the YF reported cases reported during the YF outbreak, Darfur region, 2012–2013 (*n* = 844).

	Variable	Frequency	Percent
**Age**	0–1.9	7	0.8
	2–4.9	17	2
	5–14.9	157	18.6
	15–29.9	462	54.7
	30–44.9	144	17.1
	45 & more	57	6.8
**Sex**	Male	655	77.6
	Female	189	22.4
**Residence**	North Darfur	178	21.1
	Central Darfur	434	51.4
	West Darfur	148	17.5
	South Darfur	81	9.6
	East Darfur	3	0.4
**Occupation**	Miner	93	11
	Herdsmen	159	18.8
	Farmer	160	19
	Student	32	3.8
	Other	56	6.6
	Unknown	344	40.8
**Vaccination status**	Vaccinated	67	7.9
	Not vaccinated	650	77
	Unknown	127	15.1

The overall attack rate (AR) was 10\100 000 and crude fatality rate (CFR) 20.3%. The most affected state was Central Darfur where the AR reached 41\100 000 and the highest CFR was reported from E. Darfur (66.7%) followed by S. Darfur (33.3%). Thirty five localities were affected in the five Darfur states. Alsereef locality in N. Darfur was the most affected one with AR of 234.8\100 000 **(****Table 2****)**.

**Table 2.** YF attack rate and case fatality rate in the affected localities, (*n* = 844).

State	Locality	Population	AR(cases\100 000)	CFR (%)
**C. Darfur**	Azom	45215	161.5	32.9
	Nertati	138 220	73.1	23.8
	WadiSailh	173 730	33.4	24.1
	Zaligi	305 320	49.5	19.2
	Mokgar	97110	18.5	0
	Bandasi	52140	24.9	7.7
	Om Dokhon	134830	12.6	17.6
	Rokoro	120615	5.8	14.3
**Total**		1067180	41	21.9
**S. Darfur**	Kass	211 063	11.8	56
	Nyla	561 162	2	9.1
	ShargAlgabal	184 962	4.9	77.8
	Mersheng	203 929	1.5	33.3
	RehaidAlbardi	297 624	2	16.7
	Kabam	305 367	2.6	12.5
	Ad Alfersan	273 873	4.4	8.3
	Alsalam	98 628	4.1	25
	Shataia	42314	2.4	0
	Tols	129001	0.8	0
	Belail	213719	0.5	0
**Total**		3322 465	2.4	33.3
**W. Darfur**	Algenina	301475	9	22.2
	Alkrenik	74 875	68.1	13.7
	Baeda	346 525	10.1	28.6
	Habeela	48 350	37.2	66.7
	Forbranga	69290	13	0
	Serba	66790	7.5	20
	Jabal Moon	43915	6.8	0
**Total**		992915	14.9	24.3
**N. Darfur**	SarafOmra	128 386	6.2	12.5
	Alsereef	70 261	234.8	4.8
	Alfashir	620 349	0.3	0
	Alwaha	34788	2.9	0
	Kabkabia	213483	0.9	0
	Meleet	160828	0.6	100
**Total**		2336201	7.7	5.6
**E. Darfur**	Yasin	87 801	1.1	0
	Asalaia	87 901	1.1	100
	Aldain	81261	1.2	100
**Total**		765336	3.4	66.7

Regarding the blood samples taken from reported cases, 134 samples (15.9%) were tested for YF, of which only 48 (35.8%) were found positive by either PCR or IgM specific antibodies or by both. However, 655 (77.6%) of the reported cases were considered to have the disease based on the epidemiological link. The remaining 55 cases had no samples or their samples had been rejected and they were not epidemiologically linked to confirmed cases. Eighty four reported cases with jaundice tested for hepatitis E infection, of which 37 samples (44%) were found positive **(****Table 3****)**.

**Table 3.** Distribution of lab results according to diagnosis yellow fever, HEV and both (the percentages calculated from reported cases = 844).

Diagnosis	Frequency	Percent (from total reported cases = 844)
**YF confirmed cases**	48	5.7%
**YF epidemiologically linked**	655	77.6%
**HEV confirmed cases**	37	4.4%
**Concurrent infection for YF and HEV**	13	1.5%

**Figure 2.**
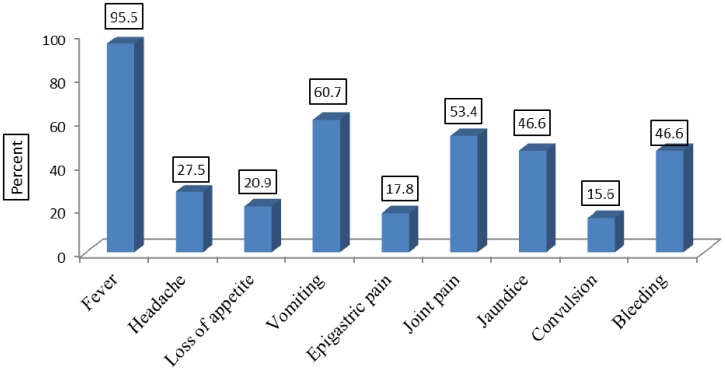
Symptoms associated with the YF reported casesduring the outbreak in Darfur region, 2012–2013 (*n* = 844).

The majority of reported cases (95.5%) presented with fever, 60.7% had vomiting and about half of cases (53.4%) developed joint pain. About 36% of reported cases have bleeding per mouth, bleeding per nose constituted 20% and about (19%) of cases had bleeding from both sites. Symptoms associated with reported cases were demonstrated in **(****Figure 2****)**.

The entomological indices calculated for each locality during outbreak have been demonstrated. They were not significantly correlated with attack rate (AR) or Case Fatality Rate (CFR) of YF calculated per localities. The highest household index (HI) and container index (CI) were recorded from Wadi Salih (28%, 14%) and Mokgar (26%, 17%) localities. All indices were highest in C. Darfur state (AR = 41, HI = 17.9% and CI = 10.6%). The household index in Central and West Darfur states was as high as 17.9 and 11.9, respectively **(****Table 4****)**.

**Table 4.** Distribution of the entomological indices and YF attack rate recorded in the affected localities, Darfur region, 2012–2013 (*n* = 8541 houses inspected).

	Locality	No. Houses inspected	Household index (%)	Container index (%)	Pupal demographic index	Breteau Index	YF AR
**C. Darfur**	Azom	70	4.3	2	0	5.7	161.5
	Bandasi	200	10	6.6	0.009	16	24.9
	WadiSalih	50	28	14.2	0.016	34	33.4
	Mokgar	220	26.4	16.9	0.029	40	18.5
	Nertati	140	20	8.2	0.022	20	73.1
	Omdokhon	200	20	9.5	0.028	24	12.6
	Zalingi	240	16	13	4.3	30	49.5
**State**		1120	17.9	10.6	2.5	25.7	41
**S. Darfur**	Ad Alfersan	500	3	1.3	0.003	3.8	4.4
	RehaidAlbardi	500	4.4	2.7	0.009	7.8	2
	Kass	540	4.6	1.7	0.01	5.2	11.8
	Nyla	1411	4.1	2.2	0.003	8.8	2
	Tlos	480	3.8	1.6	0.003	5	0.8
**State**		3431	4	2	0.5	6.8	2.4
**W. Darfur**	Algenina	894	20.5	7	0.026	31.1	9
	Baeda	240	5	0.9	0	2.5	10.1
	Forbaranga	1080	16.5	1.7	0.016	3.4	13
	Habeela	546	13	6.2	0.038	15.4	37.2
	Krenik	1230	2.5	1.2	0.015	2.8	68.1
**State**		3990	11.9	3.9	.02	1.5	14.9

## 4. Discussion

The estimated attack rate (AR) of the outbreak was 10\100 000. It was lower than AR recorded in YF outbreaks that had occurred in Kenya (1992–1993) and Uganda (2010) which were associated with attack rates of 27.4/100 000 and 13/100 000, respectively [9],[10]. The crude fatality rate (CFR) of current epidemic (20.3%) was double that occurred in the Nuba Mountains outbreak, but it was exceeded by the fatality rate of Imatong outbreak (29.6%), South Kordofan (27%), Nigeria in 1969 (40%) and Uganda in 2010 (24.9%) [10],[12]–[14]. East Darfur reported the highest fatality rate because 3 cases were reported from this state Two of them died. The fatality rate was very high in Sharg Algabal and Habeela localities which recorded 77.8% and 66.6% respectively. It could be attributed to deteriorated health services in these localities.

The most affected state was Central Darfur where the AR reached 41\100 000 and the highest CFR was reported from East Darfur (66.7%) followed by South Darfur (33.3%). Thirty five localities were affected in the five Darfur states. The number of cases may be higher because the cases started to appear in this area many miners left. In addition, not all cases were registered which lead to under reporting of the outbreak. High CFR indicated the inclusion of high percentage of severe cases that suffered toxic phase, which could be a consequence of late presentation of YF cases.

Data about residence of reported cases were as follow; Central Darfur state was the highest, whereas the lowest percentage was reported from East Darfur. Most of the cases were males (77.6%) and almost half of the cases (54.7%) aged between 15–29 years old. This was similar to the earlier outbreak in South Kordofan where 60.5% of cases were also males and third of suspected cases were at age group of 15–29 years old (unpublished data). Many studies found adult males more affected during YF outbreaks, because males more mobile at daytime and usually come in close contact with Aedes egypti which most commonly bite at dawn and dusk. Ecological characteristics of Darfur region, that characterized by the presence of valleys surrounded by many forests, in addition to many monkeys lives in the area which lead to available YF cycle, these could be the source of infection. Unfortunately there was no epizootic survey done to investigate sylvatic transmission among monkeys in the affected area. At the locality level, there was no significant association found between entomological indices collected from houses with attack or fatality rates of reported cases. In Darfur, adult males could be more affected because they were more likely to work as farmers or herdsman. In contrast, during the outbreak occurred in Gambia (1978–1979) and Ghana (1969–1970), children under 15 years were most affected because many African countries stop the routine mass YF vaccination campaign since 1960s [3],[15].

In the line list of YF, there was no occupation data for 41% of reported cases. The data about the occupation is important because it can give clue to the source and nature of the disease. In the current outbreak, farmers and herdsmen were more affected than others. Based on the date of symptoms onset, the disease was reported first within herdsmen (among who were the 2 index cases).There was a significant statistical association between fatality rate and occupation (chi-square 22.320, *P* = 0.0001) where the fatality rate was the highest among herdsmen (26.2%). It could be due to remote area of works which made them inaccessible to the health services.

As a result of late presentation of YF reported cases, many cases attended with severe clinical manifestations like bleeding, jaundice and convulsion accounted for (46.6%), (46.6%), and 15.6%, respectively. It could also be attributed to delay in diagnosis because the mild form of YF usually associated with nonspecific symptoms which can easily be confused by malaria and other endemic diseases.

Therefore, a question like “why headache, joint pain, epigastric pain and jaundice were associated with decreased fatality?” was arise. The answer is, this could be a result of the presence of another less fatal disease with similar clinical features captured by surveillance system which was not committed to already broad case definition of suspected cases.

When we reviewed the laboratory investigation of 84 reported cases which have jaundice, we found that 80 cases (95%) of them were tested for hepatitis E infection and 33 cases(41.3%) was found positive. Data also showed that more than half of reported cases with jaundice, who were found negative for YF, were belonged to North Darfur state. This state was affected by hepatitis E epidemic at the same year 2012 (unpublished data). Hepatitis E affects most commonly the same age group (15–30) and has similar clinical features (jaundice, joint pain and abdominal pain). The fatality rate among those, who were YF confirmed negative cases with jaundice, was found very low 3.3% (it is similar to hepatitis E fatality rate in the literature) [16]. In addition to (1.5%) of reported cases which had YF and HEV co-infection which complicated the situation. In Uganda YF outbreak 2010, the same phenomenon was observed where suspected cases included cases of ongoing hepatitis E epidemic in northern Uganda [10].

The entomological indices calculated for each locality during outbreak demonstrated that the highest house index (HI) and container index (CI) were recorded from Wadi Salihand Mokgar localities. All indices were highest in C. Darfur state (House Index = 17.9% and Container index = 10.6%). The house index in Central and West Darfur states were as high as 17.9 and 11.9, respectively. On statistical analysis, when ANOVA with Tukey post hoc test were used to detect significant differences between affected states regarding entomological indices. Central Darfur state was significantly higher in all studied entomological indices (except Pupal Demographic Index which was also higher but not statistically significant). The household index (HI) in Central and West Darfur states were significantly higher than South Darfur constituted 17.9% and 11.9% respectively; it was exceeded the (threshold) level of YF transmission (<5%).

At the locality level, the entomological indices calculated for each locality during the outbreak period were not significantly correlated with AR or CFR for that locality. Since the collected entomological indices are indicators for urban cycle transmission, the absence of those correlations could support the evidence of sylvatic transmission. In other hand, when cross correlation function was used for pooled data of all localities, the number of daily reported cases (epidemic curve) showed moderate significant correlation (*r* = 0.46, 95% CI = 0.05 to 0.87) with the daily means of entomological indices (CI and HI) (Figures 4). However the correlation was at lag (−7), which indicted that increase, in entomological indices (CI and HI) was followed by increase in number of reported cases 7 days later. This lagging period could be equivalent to the incubation period of YF which ranged from 2–6 days [17]. It could be an indication of YF transmission which propagated through urban cycle when infected mosquitoes bitted new victims and spread the infection. Presence of both types of transmission could be interpreted by presence of intermediate cycle “in which monkeys and humans can be infected by mosquitoes that breed both in wild and around households” as in an outbreak of yellow fever (YF) occurred in Senegal during October 1995 [18].

The study concluded that important risk factors of the current YF outbreak included male gender, young adults' age, herdsman or mining occupations and low vaccination coverage. Fatality rate was higher among unvaccinated group, female gender and elderly age group. Central Darfur state was significantly higher in all studied entomological indices than other states. Entomological indices have no direct correlation with morbidity indices calculated for reported cases at locality level, but have moderate correlation at regional level which could represented a state of intermediate transmission cycle.

The broad case definition and low commitment to operational case definition used during the outbreak lead to including of cases belonging to on-going hepatitis E outbreak. Political unrest with fragile administrative structure and weak surveillance system added to the complicated picture of the situation which increases the chance of occurrence of other YF outbreaks in the future.

The limitations of the study were the scarcity of laboratory reagents in national public health Laboratory (NPHL) resulted in delay of laboratory confirmation. It was officially notified by the National Public Health Laboratory on 26 October 2012. For the same reason, fewer numbers of blood samples were tested for YF; when one or two cases reported positive from a locality. The latter was affected and no further blood samples were tested for the suspected cases reported from such locality, but they were regarded as positive cases depending on the epidemiological link.
